# Tumor Inflammation Confounds Radiographic Determination of Extranodal Extension in HPV-Associated Head and Neck Squamous Cell Carcinoma

**DOI:** 10.3390/cancers18183030

**Published:** 2026-09-18

**Authors:** Sri Vemulamanda, Yasine Mirmozaffari, Alan H. Zhao, Noman Khan, Wendell G. Yarbrough, Natalia Issaeva, Benjamin Y. Huang, Travis P. Schrank

**Affiliations:** 1 Department of Otolaryngology/Head and Neck Surgery, UNC School of Medicine, Chapel Hill, NC 27599, USA; yas@med.unc.edu (Y.M.); dell@med.unc.edu (W.G.Y.); natalia.isaeva@med.unc.edu (N.I.); 2 Department of Radiology, University of Pennsylvania, Philadelphia, PA 19104, USA; alan_zhao@med.unc.edu; 3 Department of Radiology, University of North Carolina, Chapel Hill, NC 27599, USA; noman.khan2@unchealth.unc.edu (N.K.); benjamin_huang@med.unc.edu (B.Y.H.); 4 Lineberger Comprehensive Cancer Center, UNC School of Medicine, Chapel Hill, NC 27599, USA; 5 Department of Pathology and Laboratory Medicine, UNC School of Medicine, Chapel Hill, NC 27599, USA

**Keywords:** extranodal extension, HPV-associated HNSCC, oropharyngeal squamous cell carcinoma, bioinformatics, tumor inflammation, RNA sequencing, machine learning

## Abstract

**Background/Objectives**: Pathologically identified extranodal extension (pENE) predicts outcome and guides the intensity of adjuvant therapy in HPV-associated HNSCC. ENE determined by imaging (iENE) is a criterion for upstaging HPV+ HNSCC in the AJCC Cancer Staging System, 9th edition; however, its prognostic value is less than that of pENE, varies between studies, and the correlation between pENE and iENE is less than optimal. This study explores biological characteristics of tumors that can decrease the predictive value of iENE. **Methods:** Pre-treatment CT scans from patients with HPV-associated HNSCC were evaluated for iENE status. RNA-Seq data from primary tumor samples were analyzed using edgeR v4.6.2 and transcriptional differences were categorized by ssGSEA. Machine learning was applied to refine gene expression changes associated with iENE and survival was analyzed with log-rank tests. **Results:** Recurrence-free survival did not differ between patients with and without iENE. Both ML-ENE status and enrichment for inflammatory signaling were associated with improved RFS (*p* < 0.01 and *p* < 0.0001). When stratified by the ML-ENE signature or the inflammatory signature, iENE status did not further separate outcomes, indicating that its prognostic information was largely captured by these gene sets. These stratified comparisons were exploratory and limited by subgroup size. **Conclusions:** These analyses suggest that tumor biology associated with an inflamed tumor microenvironment may confound determination of ENE by imaging and that accounting for tumors with inflammatory signaling could improve the correlation of iENE with outcomes.

## Simple Summary

Extranodal extension (ENE) is defined as the spread of a tumor beyond the wall of an involved lymph node. When determined pathologically, ENE is a negative prognostic marker for HPV-associated head and neck squamous cell carcinoma and helps to inform the intensity of adjuvant therapy. When ENE is determined by imaging (iENE), it does not correlate perfectly with pathologically defined ENE (pENE) and its prognostic value is less well established. One potential explanation of the discrepancy between iENE and pENE is the radiographic appearance of inflammation in and around a lymph node that can mimic imaging characteristics of ENE. In this study, we used RNA sequencing and machine learning-based classification to characterize the biologic characteristics of the primary tumor underlying radiographically determined ENE. We found that tumors labeled iENE-positive were enriched for inflammation-related gene expression in a portion of tumors. These findings support the hypothesis that an inflamed tumor microenvironment may confound the radiographic assessment of ENE. Because tumor inflammation has been correlated with improved patient outcomes, the association of inflammation with iENE may decrease how well iENE correlates with poor patient outcomes. Consistent with this possibility, survival differences in our cohort tracked with inflammatory gene expression rather than with iENE itself.

## 1. Introduction

Head and neck squamous cell carcinoma (HNSCC) is the seventh most common cancer worldwide; its incidence continues to rise despite declining tobacco use over the past two decades [1,2]. This increase is largely attributable to high-risk human papillomavirus (HPV), a well-established driver of oropharyngeal tumorigenesis [3]. HPV-positive (HPV+) HNSCC represents a clinically and molecularly distinct entity compared with HPV-negative disease, which is primarily associated with traditional carcinogens such as tobacco [4]. Compared to HPV-negative HNSCC, patients with HPV+ disease present at a younger age and have improved outcomes, reflecting enhanced responsiveness to conventional therapies, including chemotherapy and radiation [5,6]. Nevertheless, approximately 25–30% of patients with HPV+ HNSCC experience disease progression [7,8,9]. Among patients with nodal metastases, extranodal extension (ENE), defined as the extension of tumor cells beyond the lymph node capsule into surrounding soft tissue, strongly associates with increased rates of locoregional recurrence, distant metastasis, and reduced overall survival when diagnosed by histopathology (pathological ENE or pENE) [10,11,12]. More recently, assignment of ENE using computed tomography (CT) and magnetic resonance imaging (MRI) has been increasingly explored [11], since it does not require the surgical excision of nodes. ENE determined by imaging (iENE) has been studied to determine its correlation with pENE and with patient outcome markers. Although multiple studies have examined the relationship between iENE and pENE, the reported correlation ranges vary widely. For CT, sensitivity ranges from 59% to 93%, and specificity ranges from 42% to 70% across studies [13]. iENE’s prognostic value as an independent biomarker has gained increasing recognition, resulting in its incorporation into the AJCC Cancer Staging System, 9th edition staging guidelines, although its optimal application continues to be explored [14,15]. Given that pENE is more strongly correlated with a poor outcome than iENE and the role of pENE in guiding escalation of adjuvant therapy intensity, methods to more tightly correlate iENE as a reliable surrogate for pENE could further strengthen its clinical utility [16]. Efforts to use iENE as a predictor for pENE have had mixed results, with some studies reporting sensitivities as low as 44–56% [17,18]. More recent approaches incorporating machine learning-based image analysis have shown more promising results, suggesting that such methods may improve iENE’s predictive value [19]. Taken together, iENE’s clinical role requires further exploration to understand its value and implement it most effectively.

A potential explanation for the prognostic variability of iENE is inflammation of the involved lymph nodes. Inflammatory changes in and around a node closely resemble the appearance of low-grade ENE on imaging, including indiscrete nodal margins, perinodal fat stranding, and irregular capsular enhancement, such that nodal inflammation may be misread as capsular invasion and inflate iENE assignment [20,21,22,23,24]. Inflammation is itself highly variable in HPV-associated HNSCC, where greater inflammation and its associated immune infiltration are widely regarded as positive prognostic factors [25,26,27,28]. Given that inflammation and ENE carry opposing prognostic associations, the radiographic overlap between them may diminish the correlation of iENE with outcome.

Despite in-depth exploration into the clinical relevance of ENE, the molecular mechanisms underlying the invasive phenotype in HPV+ HNSCC leading to ENE remain poorly defined. In this study, we used RNA-Seq to identify biological pathways that correlate with iENE. We hypothesized that tumor characteristics correlating with iENE may include both pathways associated with tumor aggressiveness and pathways that confound radiographic assessment such as inflammation [20,21,22,23,24]. To specifically interrogate this inflammatory signal, we used a co-expressed set of inflammatory genes that we previously identified in HPV+ HNSCC using unbiased weighted gene co-expression network analysis (WGCNA) [29]. This gene set represents a discrete gene module enriched in tumors with high inflammation and gross immune infiltration, hereafter referred to as the HPV+ HNSCC inflammatory tumor microenvironment (TME) signature. Utilizing machine learning, we aimed to distinguish the confounding inflammatory signal from transcriptional changes associated with true extranodal extension, resulting in a more refined transcriptomic profile of iENE.

## 2. Materials and Methods

### 2.1. RNA Isolation, Library Preparation, and Sequencing

Formalin-fixed paraffin-embedded (FFPE) tissue samples were sent to the UNC Lineberger Comprehensive Cancer Center (LCCC) Translational Genomics Lab (TGL) for RNA isolation using the Maxwell 16 MDx Instrument (Promega, Madison, WI, USA; AS3000) and the Maxwell 16 LEV RNA FFPE Kit (Promega, Madison, WI, USA; AS1260) following the manufacturer’s protocol (Promega, Madison, WI, USA; 9FB167). Hematoxylin and eosin (H and E) stained slides were used for pathological identification of the tumor area, from which RNA was extracted using macro-dissection from unstained slides. Total RNA quality was measured using a NanoDrop spectrophotometer (Thermo Fisher ScientificWaltham, MA, USA; ND-2000C) and a TapeStation 4200 (Agilent, Santa Clara, CA, USA; G2991AA). Total RNA concentration was quantified using a Qubit 3.0 fluorometer (Life Technologies, Thermo Fisher Scientific, Waltham, MA, USA; Q33216). Libraries were prepared with Illumina TruSeq Stranded Total RNA with the Ribo-Zero protocol (Illumina, San Diego, CA, USA). Libraries were sequenced on an Illumina HiSeq2500 sequencer (Illumina, San Diego, CA, USA). Paired-end read data, with read lengths of 75 bp, were collected. Archival tissue was sequenced and a corresponding chart review was performed after IRB approval was obtained (UNC IRB 15-1604).

### 2.2. Patient Cohort and Clinical Data

The retrospective cohort consisted of 81 HPV+ HNSCC patients treated at UNC hospital for whom tumor FFPE specimens were available. Detailed characteristics for this cohort are outlined in Table S1. RNA-Seq was performed on primary tumor FFPE specimens, whereas iENE is an imaging feature of affected metastatic lymph nodes. These data have been deposited at dbGaP (Study Accession: phs002935.v1.p1, “Transcriptomic Profiling of Oropharyngeal Squamous Cell Carcinoma”). No new sequencing data were obtained for this study. Related clinical data were previously obtained from a retrospective chart review and de-identified after obtaining IRB approval. The majority of patients were treated with definitive chemoradiation, the standard of care at the time of this archival cohort (Table S1); therefore, pENE data were largely unavailable.

To determine HPV16 status, viral gene expression was quantified using Salmon and the HPV16 A1 genotype, RefSeq NC_001526. HPV16 reads demonstrated a bimodal distribution; visual thresholding was performed to select HPV-positive tumors. Only tumors with a primary anatomical site of the tonsil or base of the tongue were included. All other tumor sites and carcinoma of unknown primary were excluded.

iENE status for the primary analyses was determined from the clinical radiology report. In the secondary analysis, a board-certified neuroradiologist and a second-year neuroradiology fellow independently re-reviewed available pre-treatment CT scans (scans with no previous neck radiation, neck dissection, or chemotherapy). Pre-treatment CT images and the CT report were retrievable for 61 of the 81 patients; the remaining 20 had a pre-treatment CT report available but no accessible images and, thus, were not included in the secondary analysis. No patients were excluded for prior neck surgery, radiation, or chemotherapy, since all scans used for both the report-based and re-review analyses were obtained before treatment. Neuroradiologists assessed established ENE correlates, including nodal central necrosis, indiscrete nodal borders, perinodal fat stranding, invasion into adjacent soft tissue structures, multiple involved lymph nodes (greater than 1 involved lymph node), lobular contour of nodes, conglomerate nodes, irregular nodal capsular enhancement, and matted nodes. Both neuroradiologists were blinded to all other data and analysis. Based on the findings, an ENE grade was also assigned per consensus criteria [23]: Grade 0, no ENE features; Grade 1, irregular or ill-defined nodal margins and/or extension into perinodal fat; Grade 2, conglomerate, matted, or coalescent nodes ± Grade 1 features; and Grade 3, clear extension into adjacent structures ± Grade 1 or 2 features. For nearly all imaging features examined, the neuroradiologists were concordant. In discordant cases, the assessment of the senior neuroradiologist was used for the final determination prior to data analysis. Cohen’s kappa was used to calculate inter-reader agreement. Radiology report-based classification of iENE was used as the primary variable due to the larger number of available reports compared to accessible pre-treatment CT scans. Additional characteristics of these patients can be found in Table S1.

### 2.3. Analysis

All differential expression analyses were performed in R v4.5.0 using edgeR v4.6.2 [30]. A total of 56,809 genes were analyzed. Of these, 22,893 survived following the filtering of low-expression transcripts. Raw counts were passed into the edgeR v4.6.2 pipeline, where they were normalized to library size and converted to logCPM. Differentially expressed genes (DEGs) were identified using the edgeR v4.6.2 glmQLfit and glmQLFTest functions. Genes were filtered for significance based on pre-specified thresholds: |log2(fold change)| > 1 and FDR < 0.05.

The machine learning workflow consisted of a single refinement cycle: (1) An initial centroid classifier was trained on all 81 samples using the 46 DEGs derived from radiology report-based iENE labels as features (logCPM values), with standard functions in the cancerclass package v1.52.0 [31]; (2) The classifier was applied to all 81 samples, identifying a subset of 53 patients labeled as confident (described in detail below); (3) DEG analysis was repeated within the “confident samples”, comparing iENE-positive with iENE-negative samples; (4) A final centroid classifier was trained on the confident samples using these DEGs as features and applied to all 81 samples and the labels it assigned are referred to hereafter as ML-ENE status; and (5) A final DEG analysis compared ML-ENE-positive with ML-ENE-negative samples and the significant DEGs were used to build the ML-ENE signature. All samples were used for feature selection and classifier training. No held-out test set or cross-validation were performed and reported AUC values are internal estimates of agreement with the initial labels. The same workflow was applied with neuroradiologist-graded ENE as the initial label for grade-based analyses. 

To define the confident samples that would be included in the training of the final model, we reasoned that a confidently labeled sample would satisfy two criteria: (i) A confidently labeled sample was clearly a member of one of two groups based on RNA-seq analysis; and (ii) A confidently labeled sample was in the same group as the majority of other samples with the same label (iENE status). To formalize these criteria for our ML workflow, we utilized the zeta score generated by the cancerclass v1.52.0 package. Zeta is a signed classification score scaled by the distance in transcriptional space between the two identified classes: −1 represents the centroid (average transcriptional state) of one class, and +1 represents the centroid of the other class. Based on the distance metric chosen (Euclidean) the range −1 to +1 represents the transcriptional difference between the average transcriptional states of the two groups. A score of 0 represents a sample that is perfectly intermediate between the two classifications. To address criterion (i), we considered samples with scores near zero to be not confidently classified by RNA. To define these intermediate and, therefore, difficult-to-classify samples, a range of −0.05 < zeta < 0.05 was chosen by visual inspection of the prediction distribution. This symmetric margin was intended to exclude samples lying essentially equidistant from the two centroids from the final model training step. To address criterion (ii), we also excluded samples where the initial iENE label and RNA-based classification were discordant. More formally, iENE-positive samples with zeta < 0.05 and iENE-negative samples with zeta > −0.05 were excluded from the training step. We note that the |zeta| > 0.05 threshold was chosen to exclude samples close to the decision boundary and was set post hoc, following inspection of the initial (pretraining) prediction distribution, considering our past success with this ML approach [32,33]. As such, this selected model boundary represents a potential source of over-fitting.

The R package clusterProfiler v4.16.0 was utilized to perform gene set enrichment analysis [34]. Genes were ranked by signal-to-noise ratio and enrichment scores were calculated for gene sets. Hallmark gene sets were downloaded from the MSigDB database; the HPV+ HNSCC inflammatory TME signature and related gene-expression modules are publicly available [29]. The HPV+ HNSCC inflammatory TME signature comprises 432 of the 464 genes in the larger wheat2 module. The 32 module genes that anticorrelated with the module median were excluded, consistent with the approach in the original report (Table S2) [29]. Over-representation analysis was performed using the enrichR v3.4 [35] and clusterProfiler v4.16.0 R packages. Genes were compared to published pathways available in the GO, KEGG, Reactome, MSigDB, and ENCODE databases. Pathways were filtered for significance if they had an adjusted *p*-value < 0.05. All ssGSEA analyses were performed using the GSVA v2.2.0 R package [36].

All survival analyses were performed using survminer v0.5.0 package [37]. Kaplan–Meier plots were generated using ggplot2 v3.5.2 [38]. Significance was determined by log-rank tests. Binary classification of enrichment for a particular gene set, determined by ssGSEA, was achieved by comparing each enrichment score to the cohort median. Firth- penalized Cox models were fitted with coxphf v1.13.4 [39]; proportional-hazards assumption was assessed with scaled Schoenfeld residuals.

## 3. Results

Reviewing our available database of HPV+ HNSCC cases with RNA-seq data, we identified 81 patients with pre-treatment CT reports, of which 21 (25.9%) were iENE-positive. Of the 81 patients with pre-treatment radiology reports, 61 also had pre-treatment CT imaging available. These images were reviewed by neuroradiologists and imaging features were correlated with reported iENE on imaging reports (Table 1). Among the radiographic features assessed, invasion into adjacent structures showed the strongest association, with radiology report-based iENE-positive status being present in 83.3% of iENE-positive cases, whereas it was present in only 28.2% of iENE-negative cases (*p* < 0.001). The presence vs. absence of an indiscrete nodal border was also significantly associated with iENE-positive status (83.3% vs. 50.0%, *p* = 0.021), as was the presence of multiple involved lymph nodes (100% vs. 75.6%, *p* = 0.025; Table 1). ENE grading similarly tracked with radiology report-based iENE status, with 83.3% of iENE-positive patients assigned Grade 3, compared to 23.3% of iENE-negative patients (overall *p* < 0.001; Table 2). Agreement between radiology report-based iENE status and neuroradiologist-determined G3-ENE status was moderate (Cohen’s κ = 0.54; Table 2). The clinical characteristics for these patients are outlined in Table S1.

**Figure 1:**
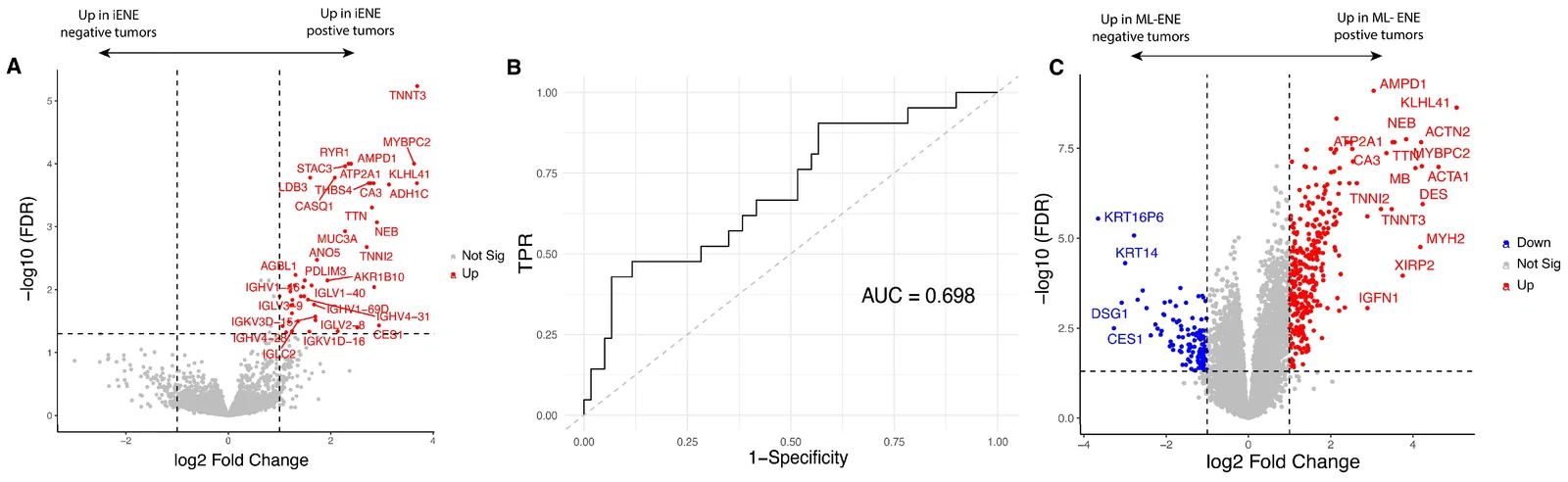
(**A**) volcano plot of differentially expressed genes (DEGs) between iENE-positive and iENE-negative samples, as designated on radiology reports. DEGs were defined as those with an FDR < 0.05 and |log_2_(fold change)| > 1 (Signifcance and fold change thresholds displayed as dashed lines on volcano plot). Upregulated genes are indicated in red. No downregulated genes met the FDR threshold; (**B**) receiver operating characteristic (ROC) curve of the ML-centroid classifier, showing true positive rate (TPR) versus 1−specificity. TPR was calculated against radiology report-based iENE labels. The area under the curve (AUC) was 0.698, reflecting classifier performance in predicting radiology report-based iENE status; and (**C**) volcano plot of differentially expressed genes between ML-ENE-positive and ML-ENE-negative predictions. Signifcance and fold change thresholds displayed as dashed lines on volcano plot. Upregulated genes are indicated in red and downregulated genes are indicated in blue.

RNA sequencing was performed to evaluate transcriptional differences between tumors with and without iENE based on radiology reports. Differential expression analysis identified 46 significantly upregulated genes in iENE-positive tumors while no downregulated genes passed the FDR threshold (Figure 1A). A full list of differentially expressed genes (DEGs) can be found in Table S3. Notably, *CA3*, whose expression correlates with epithelial–mesenchymal transition (EMT) and metastasis, was highly expressed in iENE-positive tumors [40]. Similarly, *CES1*, a well-established *NRF2* target gene and marker of oxidative stress, radiation resistance, and tumor aggressiveness, had increased expression in iENE-positive tumors [41,42,43,44]. A subset of differentially expressed genes was related to B-cell antibody production, such as *IGLV2-8*, *IGKV3D-15*, *IGHV1-69D*, and *IGLV1-40*, indicating enrichment of lymphocytes in iENE-positive samples [45,46]. Interestingly, many genes associated with sarcomere muscle function (e.g., *TNNT3*, *KLHL41*, *MYBPC2*, *NEB*, and *TNN*) were highly expressed in iENE-positive samples [47]. The absence of significantly downregulated DEGs reflects an asymmetry in fold change as well as adjusted *p*-value. A mixture model of multiple cell types with minor populations of cells in the tumor microenvironment, some with high expression of a relatively small number of genes, is an attractive explanation for this effect, in general [48]. Biological heterogeneity amongst samples in each of the two groups is another important consideration; this concept is supported by the more symmetric appearance of the differential expression volcano plot after ML-based refinement of the tumor groups (Figure 1C).

These 46 upregulated genes were used to assign ENE status from tumor expression profiles. Samples with ambiguous predictions (|zeta| < 0.05) or discordant iENE status (classifier-assigned ENE status did not match the iENE status on the radiology report) were excluded, and a refined classifier was generated using only high-confidence cases. Application of this classifier to the full cohort yielded an AUC of 0.698 (Figure 1B).

Using ENE, as classified by the machine learning (ML) gene expression classifier (ML-ENE status), a second round of analysis identified 447 differentially expressed genes meeting both FDR and expression thresholds and comprising 115 downregulated and 332 upregulated transcripts (Figure 1C). Among these, *DSG1*—whose loss has been associated with metastatic progression—was significantly downregulated in ML-ENE-positive tumors [49]. Reduced expression of *KRT14* and *KRT16P6* was observed, indicating that despite established roles in proliferation, migration, and survival, lower levels of their expression are associated with ML-ENE-positive status in HNSCC [50,51]. A full list of DEGs can be found in Table S4.

**Figure 2:**
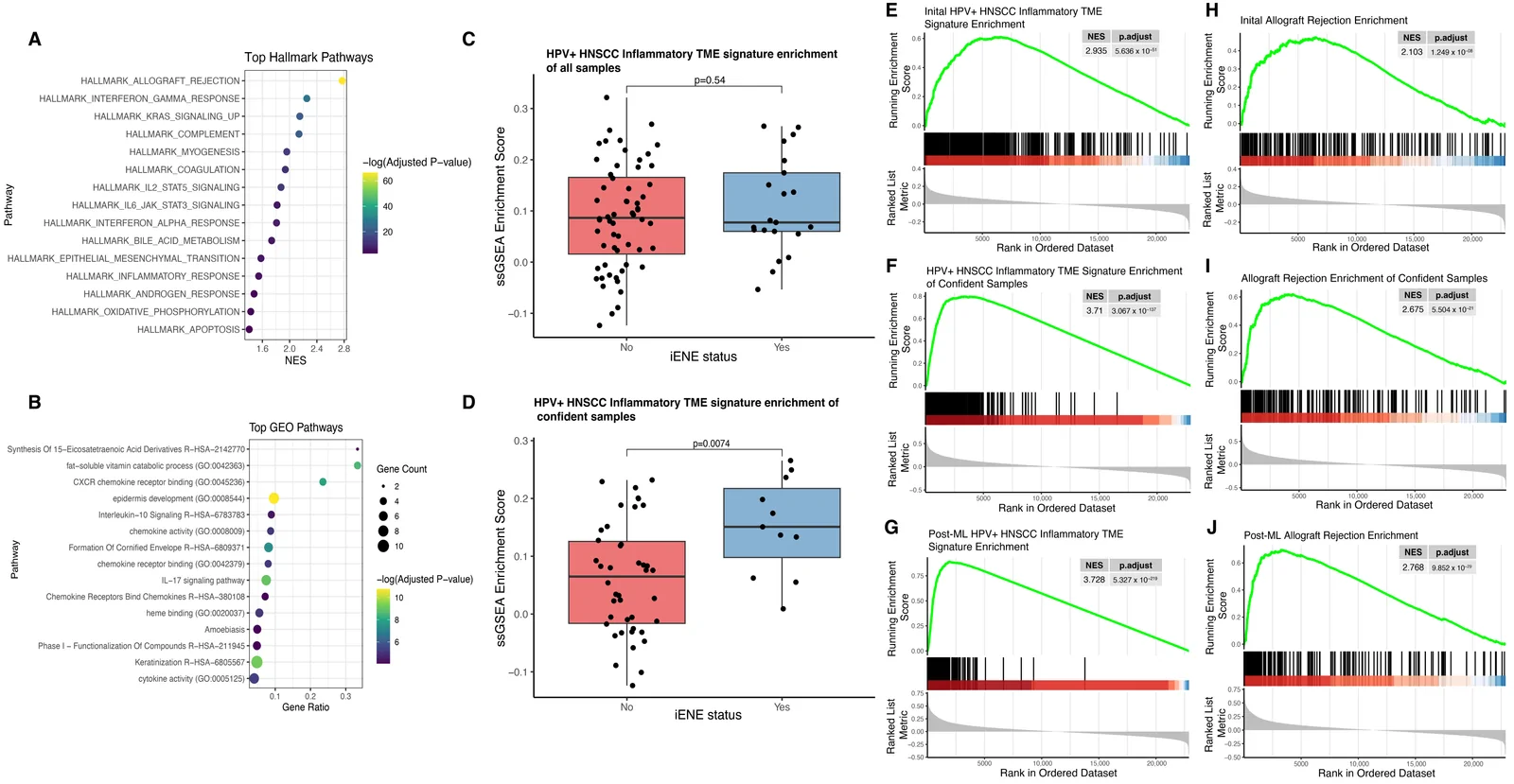
(**A**) gene set enrichment analysis of MSigDB hallmark gene sets based on differentially expressed genes (DEGs) between ML-ENE-positive and ML-ENE-negative tumors. Dot color indicates −log (adjusted *p*-value); (**B**) gene ontology (GO) analysis of DEGs derived from ML-ENE classifier. Dot size reflects the number of overlapping genes between each gene set and the DEGs, and dot color indicates −log (adjusted *p*-value); (**C**) box plot of HPV+ HNSCC inflammatory TME enrichment scores determined by single-sample gene set enrichment across all cohort samples (Mann–Whitney U test, *p* = 0.54); (**D**) box plot of HPV+ HNSCC inflammatory TME enrichment scores determined by single-sample gene set enrichment in samples with confident ML-ENE status (Mann–Whitney U, *p* = 0.0074); (**E**–**G**) gene set enrichment plots for the HPV+ HNSCC inflammatory TME gene set in DEGs from all samples stratified by iENE status (**E**), restricted to confident samples (**F**), and all samples stratified by ML-ENE status (**G**); and (**H**–**J**) gene set enrichment plots for the hallmark gene set allograft rejection in DEGs from all samples stratified by iENE status (**H**), restricted to confident samples (**I**), and all samples stratified by ML-ENE status (**J**).

Pathway analysis of differentially expressed genes between ML-ENE-positive and ML-ENE-negative tumors demonstrated significant enrichment in epithelial to mesenchymal transition and inflammation-related pathways in ML-ENE-positive tumors. Particularly striking was the highly significant identification of the allograft rejection hallmark pathway, which had a very high normalized enrichment score (NES = 2.8). The allograft rejection pathway contains approximately 200 genes that are upregulated during organ transplant rejection and broadly represent adaptive and innate immune activation and inflammation. Comparison with hallmark gene sets also revealed significant association with interferon-γ response, IL2–STAT5 signaling, IL6–JAK–STAT3 signaling, interferon-α response, and inflammatory response pathways (Figure 2A). Similarly, over-representation analysis identified enrichment in related processes such as CXCR chemokine receptor binding, interleukin-10 signaling, chemokine activity, and IL-17 signaling pathways (Figure 2B).

To determine if the ML-ENE classifier gene set overlapped with endogenously co-expressed gene modules that we previously identified in HPV+ HNSCC, we compared these gene sets [29]. Endogenous co-expressed gene sets were developed and validated across multiple independent cohorts using unsupervised weighted gene correlation network analysis identifying modules or clusters of co-expressed genes that naturally occur in HPV+ HNSCC [29]. Gene set enrichment analysis (GSEA) of differentially expressed genes based on ML-ENE status showed strong enrichment in the HPV+ HNSCC inflammatory tumor microenvironment (TME) gene signature (Figure 2G). The HPV+ HNSCC inflammatory TME signature consists of 432 genes (Table S2) and, of the 22 co-expressed gene sets, is the one most highly enriched in genes indicating immune activation or inflammation [29].

Evaluation of the centroid classifier parameters demonstrated a clear increase in single-sample GSEA (ssGSEA) HPV+ HNSCC inflammatory TME enrichment scores after restricting to high-confidence samples (those in which the initially assigned ENE status matched the radiology report-based iENE label and where the prediction met the confidence threshold, |zeta| > 0.05) (Figure 2C,D). In parallel, the normalized enrichment score (NES) increased and the adjusted *p*-value decreased across successive enrichment analyses for both the HPV+ HNSCC inflammatory TME (Figure 2E–G) and allograft rejection (Figure 2H–J) gene sets, progressing from iENE of all samples (based on the radiologist’s report), to iENE labels of confident samples, and, finally, to ML-ENE status.

**Figure 3:**
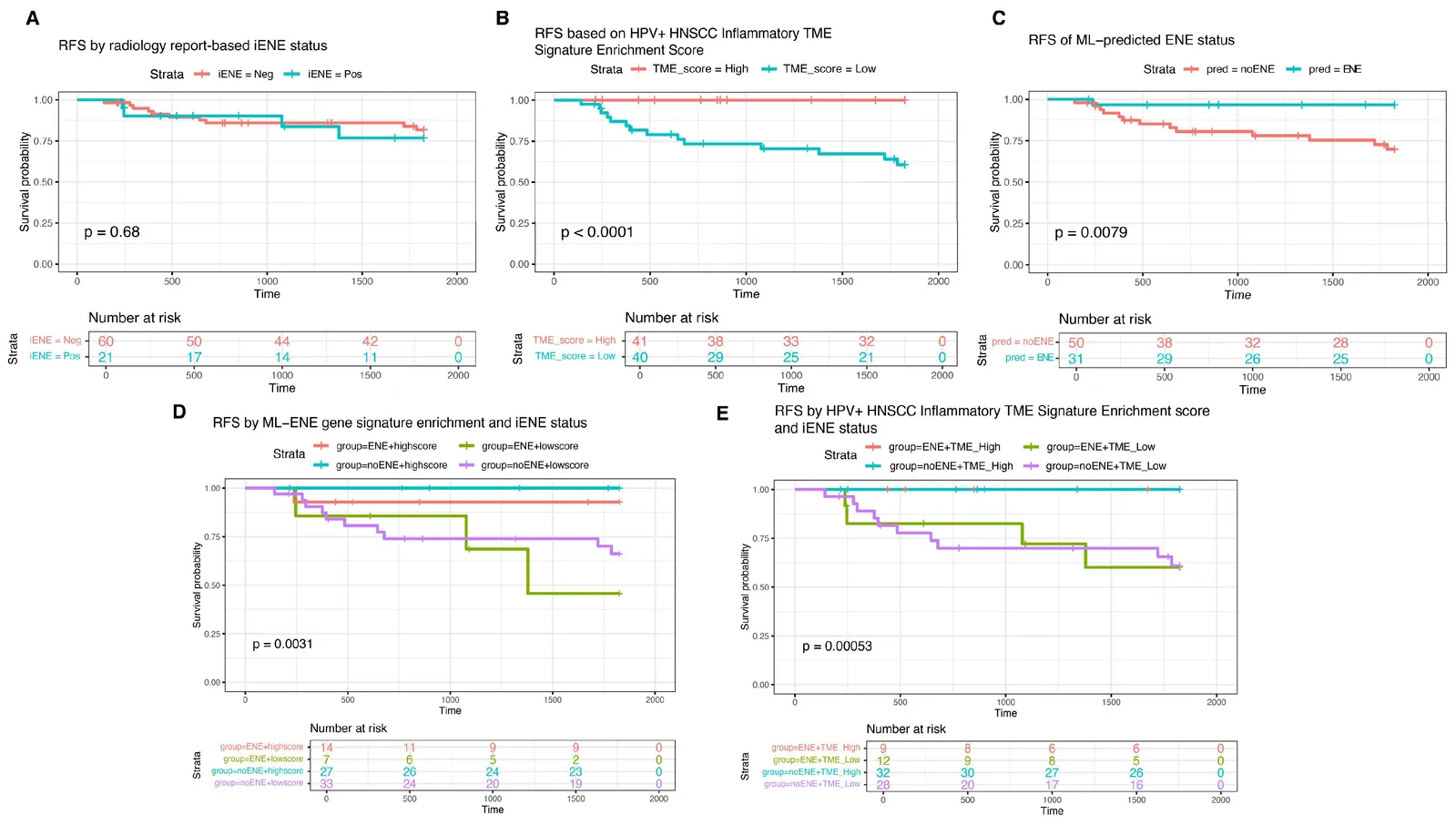
(**A**–**C**) recurrence-free survival (RFS) of the cohort stratified by radiology report-based iENE status (**A**), HPV+ HNSCC inflammatory TME enrichment score (**B**), or ML-ENE status (**C**); and (**D**,**E**) RFS was further analyzed by grouping samples according to radiology report-based iENE status and ML-ENE signature enrichment (**D**), or radiology report-based iENE status and HPV+ HNSCC inflammatory TME signature enrichment (**E**). Samples were classified as high if their enrichment score was greater than or equal to the cohort median, and low otherwise. Nominal *p*-values are shown in Kaplan–Meier plots. The number of events per strata and FDR-corrected values are available in Table S6.

Analysis of recurrence-free survival (RFS) demonstrated no significant differences between iENE-positive and iENE-negative groups based on radiology reports (Figure 3A). However, stratification by enrichment for the HPV+ HNSCC inflammatory TME signature was robustly correlated with outcome (Figure 3B), as was stratification by ML-ENE status (Figure 3C), in which defining DEGs were enriched for immune and inflammatory signaling (Figure 2). ML-ENE-positive tumors exhibited significantly longer RFS than ML-ENE-negative tumors (Figure 3C).

When the cohort was further stratified by both radiology report-based iENE status and either the ML-ENE signature (Figure 3D) or the HPV+ HNSCC inflammatory TME signature (Figure 3E), the addition of iENE status did not significantly separate outcomes within either the high- or low-enrichment groups. In contrast, both gene signatures clearly separated outcomes within the iENE-positive group: iENE-positive tumors with high enrichment for either the HPV+ HNSCC inflammatory TME signature or the ML-ENE signature showed improved RFS compared with iENE-positive tumors with low enrichment (Figure 3D,E). To test whether iENE contributed prognostic information beyond either signature, Cox proportional-hazards models were fitted containing iENE status, signature status (high versus low), and their interaction. Following adjustment for iENE, signature enrichment remained associated with RFS for both the HPV+ HNSCC inflammatory TME high group (HR 0.03, 95% CI 0.0003 to 0.26, *p* < 0.001) and the ML-ENE signature high group (HR 0.05, 95% CI 0.0004 to 0.36, *p* < 0.001), whereas iENE status was not associated with RFS after adjustment for either signature (HR 1.09, 95% CI 0.32 to 3.13, *p* = 0.88; HR 1.68, 95% CI 0.42 to 5.22, *p* = 0.42, respectively). No interaction between iENE and signature status was detected (likelihood-ratio *p* = 0.50 and 0.20), although, with 14 recurrence events, this test had limited power, and the proportional-hazards assumption was not violated in either model (Table S5). Together with the absence of a standalone iENE effect (Figure 3A), these data suggest that the prognostic information associated with iENE in this cohort is largely captured by the inflammatory TME signature and the ML-ENE signature, with no independent contribution of iENE in this cohort. Given the limited size of these subgroups, there is limited statistical power for subgroup interaction inference, and we cannot exclude an independent contribution of iENE. Larger cohorts will be needed to determine whether any residual prognostic value remains once inflammatory signaling is accounted for.

**Figure 4:**
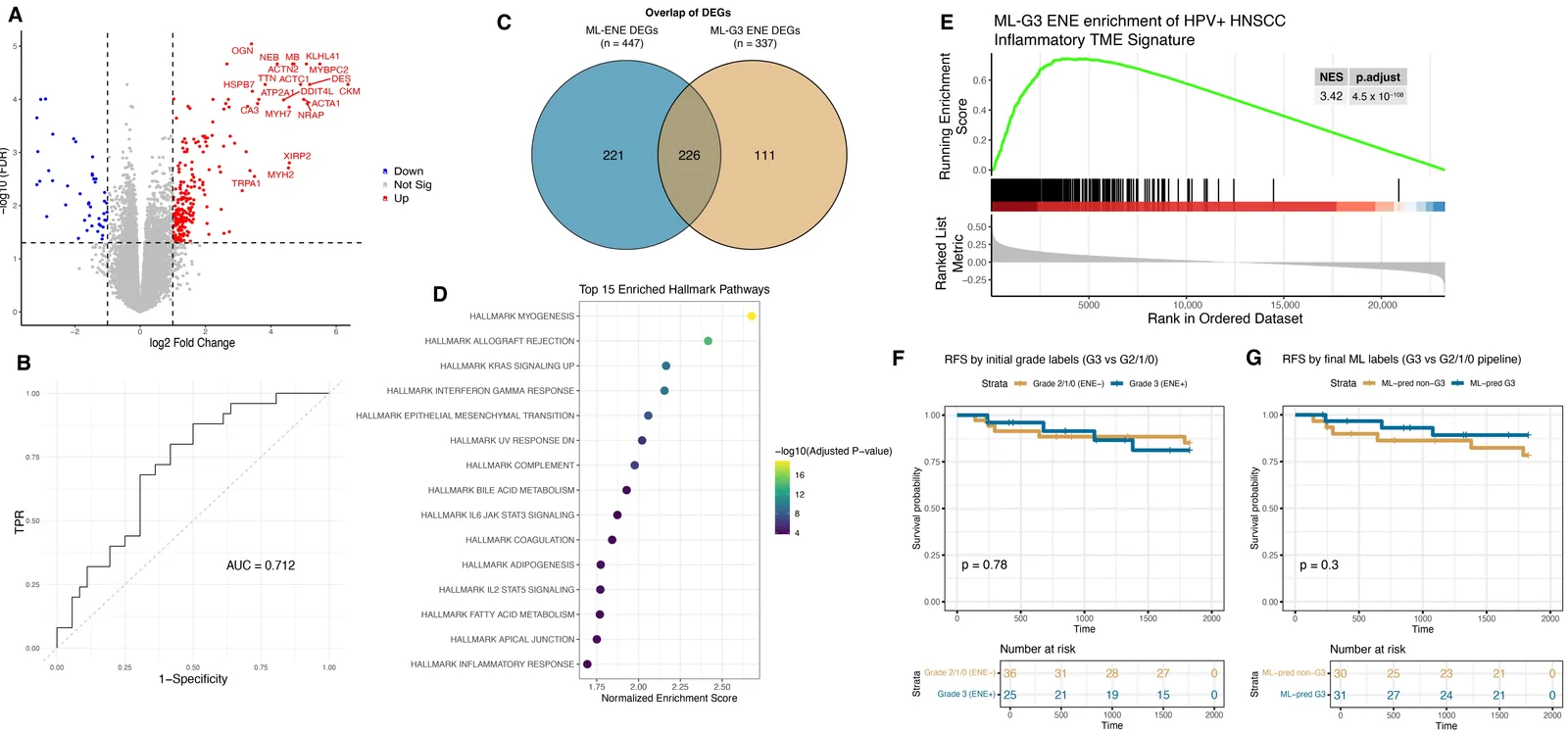
(**A**) volcano plot of differentially expressed genes between machine learning-predicted Grade 3 samples (ML-G3) and machine learning-predicted Grade 2/1/0 (ML-non-G3), with significantly upregulated genes shown in red and significantly downregulated genes shown in blue. Signifcance (FDR < 0.05) and fold change (|log_2_(fold change)| > 1) thresholds displayed as dashed lines on volcano plot; (**B**) ROC plot of final centroid classifier performance in predicting Grade 3 ENE relative to neuroradiologist classification; (**C**) overlap of DEGs found following machine learning optimization using radiology report-based iENE status (ML-ENE, *n* = 447) or neuroradiologist G3-ENE status (ML-G3, *n* = 337) for initial labeling; (**D**) gene set enrichment analysis of MSigDB hallmark gene sets based on differentially expressed genes (DEGs) in ML-G3 samples; (**E**) gene set enrichment plots for the HPV+ HNSCC inflammatory TME gene set in DEGs from ML-G3 samples; and (**F**,**G**) RFS of the cohort comparing Grade 3 ENE vs. Grade 2/1/0 based on initial neuroradiologist classification or ML-G3 ENE vs. ML-non-G3. Nominal *p*-values are shown in Kaplan–Meier plots. The number of events per strata and FDR-corrected values available in Table S6.

To determine whether the impact of inflammatory signatures on the prognostic implications of iENE would be minimized by expert radiology reading and classification of iENE, we applied the same ML-based classification pipeline using consensus neuroradiology grades as the initial labels, comparing tumors with Grade 3 characteristics of iENE to Grade 2/1/0. Grade 3 was selected due to its stringency and high concordance with radiology report-based iENE-positive status, with 83.3% of radiology report-based iENE-positive patients assigned Grade 3 (Table 1 and Table 2). Following the same machine learning-based re-classification approach outlined above, we identified 337 DEGs between machine learning-predicted G3 (ML-G3) samples vs. non-G3 (ML-non-G3) samples (Figure 4A). The centroid classifier achieved an AUC of 0.712 when predicting patients with G3 ENE compared to neuroradiologist grading (Figure 4B). Notably, 226 of 337 DEGs (67.1%) identified comparing ML-G3 vs. ML-non-G3 tumors overlapped with DEGs identified from ML-ENE-positive vs. ML-ENE-negative (Figure 4C). GSEA revealed concordant pathway enrichment in the machine learning-derived genes, whether starting from differentially expressed genes between ML-G3 vs. ML-non-G3 tumors or from differentially expressed genes between ML-ENE-positive vs. ML-ENE-negative tumors (compare Figure 2A and Figure 4D). GSEA once again highlighted immune and inflammatory pathways, showing enrichment in allograft rejection, interferon-γ response, IL6–JAK–STAT3 signaling, IL2–STAT5 signaling, inflammatory response pathways, and the HPV+ HNSCC inflammatory TME (Figure 4D,E). RFS based on neuroradiology iENE Grade G3 vs. non-G3 did not reveal a significant survival difference between G3 and non-G3 tumors (Figure 4F). Following ML-based reclassification, no significant difference in RFS was observed between tumors classified as ML-G3 vs. ML-non-G3; however, there is potentially a trend toward improved survival for ML-G3 patients vs. ML-non-G3 patients (Figure 4G).

## 4. Discussion

In this study, we sought to characterize the transcriptional landscape of iENE in HPV-associated HNSCC using RNA sequencing and machine learning-based classification. Despite ENE’s well-established negative prognostic implications when determined pathologically, our findings illustrate discrepancies in the prognostic value between iENE and the features that define it.

Our initial differential gene expression analysis comparing iENE positive vs. negative tumors based on radiology reports revealed a small subset of genes that were upregulated in iENE-positive tumors (Figure 1A). The higher expression of genes related to EMT and metastasis, such as *CA3*, correlates well with the aggressive nature of tumors with ENE, but the association of sarcomere-related genes, also upregulated in iENE positive tumors, is less well defined. Differences in sarcomere gene expression could also result from varying amounts of skeletal muscle within the macro-dissected primary tumor samples, since the base of the tongue and tonsil are both muscle-adjacent sites. Some of these genes have been implicated in HNSCC tumor progression and invasiveness, but further study is required to determine how they may impact ENE [52]. Interestingly, the comparison between iENE-positive and -negative tumors revealed a strong and highly significant enrichment of upregulated genes associated with immune activation and inflammation. The association of these inflammatory profiles with iENE was unexpected, as iENE is associated with worse outcomes and tumor inflammation is generally associated with a positive prognosis in HNSCC [14,15,25,26,27,28]. These findings raise the possibility that imaging characteristics of ENE may overlap with imaging characteristics of inflammation, and that the contamination of true ENE positive cases with cases lacking ENE—but instead having inflamed tumors—would decrease the accuracy of ENE identification by imaging and diminish the prognostic association of iENE with outcome.

To better define genes associated with iENE by avoiding genes that would not contribute to or confound classification based on iENE, we used a machine learning-based centroid classifier to identify misclassified or ambiguous samples in our dataset. The model was trained on all samples in our dataset, with initial labels based on radiology report-based iENE status. The classifier was utilized to assign ENE status on the complete cohort and identify confident samples (*n* = 53). Confident samples were then used to repeat the differential expression analysis and to train a second centroid classifier, which was applied to all 81 samples to assign ML-ENE status, yielding an AUC of 0.698 (Figure 1B). We note that only one iteration of model training was performed to avoid overinterpretation of the data. As expected, our ML model identified a moderate number of samples in which the expression profile was inconsistent with the radiology report-based label but consistent with the reported discordance between iENE and pENE [13,17].

We then performed a final differential gene expression analysis utilizing ML-ENE labels generated from our final centroid classifier (Figure 1C). When performing GSEA and over-representation analysis (ORA) on these genes, we unexpectedly found that pathways related to inflammation signaling were enriched after ML processing (Figure 2A,B). Inflammatory signatures are linked to better prognosis in HNSCC; therefore, finding these profiles enriched in ML-ENE-positive samples was surprising. To identify potential causes, we performed ssGSEA on our cohort for enrichment of the HPV+ HNSCC inflammatory TME signature. We found that, when considering all samples, enrichment for this signature does not vary, but in samples confidently classified for iENE, the HPV+ HNSCC inflammatory TME signature is significantly enriched in the iENE-positive group (Figure 2C,D). Additionally, when tracking enrichment using GSEA for the HPV+ HNSCC inflammatory TME signature and the hallmark gene set “allograft rejection”, there is a similar increase in both the normalized enrichment score and significance when moving from iENE labels to confident samples only to ML-ENE labels (Figure 2E–J). As such, we conclude that inflammatory signatures represent a prominent transcriptional feature within the iENE group, and that disentangling these from changes directly associated with extranodal extension may be key to optimizing iENE’s prognostic utility. These preliminary analyses suggest that some iENE misclassification may reflect radiologic classification of inflamed tumors as ENE-positive, which would imply that imaging characteristics of inflamed tumors overlap with those of ENE. Correlation of inflammatory tumor profiles with iENE, as identified in this study, identifies a plausible biological confounder that may impact iENE prognostic accuracy [14,15,16,17,18]. Although this study was limited by the absence of pathologic nodal classification, an additional hypothesis generated by these findings is that tumor inflammation correlating with iENE contributes to a discordance between iENE and pENE.

The hypothesis that inflamed tumors may contribute to a radiologic reading of iENE-positive is also consistent with the moderate correlation of iENE with outcome. In our cohort, radiology report-based iENE status had no significant prognostic value in predicting RFS (Figure 3A). By contrast, enrichment for the HPV+ HNSCC inflammatory TME signature and ML-ENE status were each significantly associated with RFS, with longer RFS in tumors with high signature enrichment or ML-ENE-positive status (Figure 3B,C). Because both measures reflect inflammatory signaling, this pattern is consistent with the favorable prognosis of inflamed tumors in HNSCC [25,26,27,28]. A classifier derived from iENE labels might be expected to mark tumors with worse outcomes; however, in our analysis, ML-ENE-positive tumors had improved RFS. The most likely explanation is noise in the initial iENE labels. Data labeling error or noise in classification problems have increasingly garnered attention in the field of machine learning [53,54,55,56]. In the specific case of iENE assignment by a radiologist, this data labeling (no matter how perfectly executed) is not expected to be strictly concordant with groups based on intrinsic tumor biology, even if one of two biologic groups is strongly associated with increased frequency of an iENE appearance in imaging studies. Therefore, if many iENE-positive cases are inflamed tumors whose nodes mimic ENE on imaging, a classifier trained on those labels will learn the strongest transcriptional feature shared across the positive group. In this cohort, GSEA revealed that the identified feature was related to inflammation rather than the invasive program expected of true extranodal extension. The improved RFS of ML-ENE-positive tumors, therefore, reflects the inflammatory biology captured by the classifier [25,26,27,28], not a prognostic property of ENE. Given these findings, the ML-ENE signature is more accurately described as an inflammatory signature derived from iENE-positive tumors than as a signature of ENE itself. In general, we feel that our findings support the broader concept that the conflation of phenotypic observables with biological states can lead to challenges in the interpretation of translational (paired clinical and molecular) data, and, furthermore, that specific attention to this potential issue may at times lead to novel insights. When we stratified by both radiology report-based iENE status and either of the gene signatures, iENE did not add significant prognostic separation within either enrichment group (Figure 3D,E). However, within the iENE-positive group, tumors with high enrichment for either the HPV+ HNSCC inflammatory TME signature or the ML-ENE signature had improved RFS compared with iENE-positive tumors lacking enrichment (Figure 3D,E). Therefore, we interpret these data to indicate that the prognostic information associated with iENE is largely subsumed within these inflammatory signatures rather than being contributed independently by iENE. Given the small size of the dual-stratified subgroups, these findings should be considered exploratory to generate a hypothesis that could contribute to the discordance of iENE and pENE, as well as the less robust correlation of iENE with outcome. Confirmation in larger multi-center cohorts is needed before conclusions regarding the independent prognostic contribution of iENE can be drawn.

To validate our findings using radiology report-based iENE, we examined its correlation with ENE features identified by neuroradiologists. We found that radiology report-based iENE classification shows high concordance with the identified correlates, especially invasion into adjacent structures, which is stated as one of the strongest markers for ENE (Table 1) [23]. Furthermore, we found that radiology report-based iENE status was in high concordance with neuroradiologist ENE grading, with 83.3% of samples classified as iENE-positive in the radiology report being classified as having Grade 3 ENE characteristics (Table 2). Notably, enrichment for inflammation was not significantly associated with any individual ENE correlate (Figure S1), suggesting that any confounding effect of inflammation is not driven by a single radiographic feature but may instead reflect a more diffuse process.

Beyond this clinical concordance, repeating our RNA analysis using neuroradiologist grading of iENE as the initial labels further supports the confounding potential of inflammation for iENE. Of the 337 genes differentially expressed between ML-G3 and ML-non-G3 samples, 226 (67.1%) were also differentially expressed between ML-ENE-positive and ML-ENE-negative samples (Figure 4C). Furthermore, GSEA revealed that ML-G3 samples were once again enriched for hallmark pathways of inflammation (Figure 4D,E). RFS trends similarly mirrored the radiology report-based analysis, with no significant difference in survival when comparing iENE grade alone (Figure 4F), but instead a trend toward ML-G3 patients having improved RFS compared to ML-non-G3 (Figure 4G). Taken together, this concordance suggests that the association between inflammation and iENE is consistent regardless of whether iENE is identified by general radiologists or by specialized neuroradiologists trained to identify these features. Furthermore, although prior discussion has addressed the potential for inflammation to confound low-grade (ie. Grades 1 or 2) iENE determination [24], these findings suggest that inflammation may also confound the determination of Grade 3 iENE, which is regarded as the most unequivocal evidence of extranodal extension [23].

Several limitations temper these conclusions. First, RNA-seq was performed on primary tumor tissue, whereas iENE is a feature of the metastatic lymph node. Analyzing sequencing from the primary tumor biopsy has the potential advantages of focusing the analysis on the intrinsic tumor biology and possibly decreasing the likelihood of capturing transcriptomic signals from nearby lymph node (immune) tissue. However, this approach also introduces a sampling mismatch between these two sites, which may confound the observed associations. The primary tumor transcriptional profiles may not fully reflect the biologic heterogeneity of the metastatic lymph nodes as compared to the primary tumor, and, certainly, distinct biologic features at metastatic sites could feasibly produce the iENE appearance. In matched primary and nodal metastases from OPSCC, lymphoid infiltration was concordant between sites in HPV/p16-positive tumors while myeloid populations diverged [57]; however, we cannot identify studies that have directly compared inflammatory gene expression between matched primary tumors and nodal metastases in HPV+ OPSCC. Thus, the extent to which primary tumor inflammation mirrors the nodal microenvironment requires further exploration. Directly testing to determine if inflammatory signaling in the primary tumor is concordant with that in nodal metastases, and whether it contributes to iENE calling and to a mismatch between pENE and iENE, will require paired primary and nodal transcriptomic data with matched nodal imaging.

Second, pENE data were not available for our cohort. All but two patients were treated with definitive chemoradiation rather than upfront surgery with neck dissection (Table S1); therefore, iENE, the ML-ENE signature, and pENE could not be compared directly; comparing all three in the same patients would clarify the biological and prognostic relationships among these measures.

Third, this is a retrospective, single-institution cohort of modest size. To our knowledge, it represents one of the largest gene expression analyses of iENE-positive versus iENE-negative HPV-associated HNSCC. However, the limited number of cases and recurrence events constrained multivariate analysis and interaction testing; therefore, we could not determine whether additional tumor or patient characteristics improved the correlation of iENE with outcome, nor whether iENE retains a prognostic value independent of the inflammatory signaling that dominates in this dataset.

Fourth, the centroid classifier was applied within the same cohort without a held-out test set or cross-validation. As such, the classifier should be viewed as an exploratory analysis characterizing transcriptional signatures associated with iENE and biological hypothesis generation, rather than a validated model to predict iENE in patients moving forward. Similarly, our use of confident samples as the basis for the refinement step introduces selection bias and circularity (limited by the single iteration of training) that may have amplified the confounding signal present in the original labels. External validation in an independent cohort would be needed to address these limitations.

Fifth, treatment was not uniform across the cohort and patient-reported outcomes were not collected. Confounding patient characteristics, such as pre-treatment infection, extent of nodal necrosis, and biopsy-related inflammation, were also not systematically recorded; thus, a sensitivity analysis excluding cases with these features was not possible and remains a direction for future work.

## 5. Conclusions

In conclusion, these analyses identify an inflammatory gene expression profile in a subset of iENE positive tumors and support the hypothesis that tumor inflammation may contribute to imaging features that overlap with those of iENE. Consistent with this, iENE status alone was not prognostic in our cohort; prognostic differences were, instead, captured by inflammatory gene expression signatures found in the primary tumor. These findings present an opportunity to refine iENE interpretation by improving guidelines to better distinguish radiographic features of ENE from those associated with inflammation, which may improve the correlation of iENE with patient outcomes. Given the recent incorporation of iENE into clinical staging in the AJCC Cancer Staging System, 9th edition, which applies to patients staged without neck dissection [58], accounting for inflammatory confounders could enhance prognostic accuracy and guide more effective treatment planning. The ML-ENE signature was correlated with RFS and its signal was closely tied to tumor inflammation. Whether iENE carries prognostic information that would enhance imaging classifiers will require validation in larger cohorts. Continued exploration into the confounding nature of inflammation may, nonetheless, enable a more accurate extraction of prognostic information from the radiographic changes associated with iENE.

## Figures and Tables

**Table 1:** Prevalence of imaging features stratified by radiology report-based iENE status *.

Feature	Samples Available	iENE+	iENE−	*p*-Value	Inter-Reader Agreement (κ)
Invasion into adjacent structures	57	15/18 (83.3%)	11/39 (28.2%)	0.000143	1.00
Indiscrete nodal border	60	15/18 (83.3%)	21/42 (50%)	0.0214	1.00
Multiple involved lymph nodes (>1 involved lymph node)	63	18/18 (100%)	34/45 (75.6%)	0.0253	1.00
Lobular contour	60	14/18 (77.8%)	21/42 (50%)	0.0525	0.97
Perinodal fat stranding	59	16/18 (88.9%)	27/41 (65.9%)	0.111	0.92
Matted nodes	61	14/18 (77.8%)	27/43 (62.8%)	0.372	0.96
Central hypodense area	60	14/18 (77.8%)	34/42 (81%)	0.74	1.00
Indiscrete primary tumor border	50	7/14 (50%)	21/36 (58.3%)	0.753	1.00
Irregular capsular enhancement	60	11/18 (61.1%)	27/42 (64.3%)	1	1.00

* Correlation between imaging features assigned by two independent neuroradiologists, and radiographic status as determined by radiology report. Fisher’s exact test was used for statistical analysis. Samples with inconclusive imaging features were excluded from calculations. Inter-reader agreement was calculated as Cohen’s kappa (κ).

**Table 2:** Distribution of neuroradiologist ENE grade by radiology report-based iENE status *.

ENE Grade	iENE+	iENE−	Inter-Reader Agreement (κ)
Grade 0 (no ENE features)	2/18 (11.1%)	8/43 (18.6%)	1.00
Grade 1 (perinodal fat invasion or irregular nodal margins)	0/18 (0%)	4/43 (9.3%)	0.65
Grade 2 (conglomerate or matted nodes)	1/18 (5.6%)	21/43 (48.8%)	0.96
Grade 3 (invasion into adjacent structures)	15/18 (83.3%)	10/43 (23.3%)	1.00

* Correlation between iENE status is based on radiology reports and ENE grades assigned per criteria outlined in Henson et al. [23]. Overall p-value for grade distribution by iENE status = 8.91 × 10^−5^ by Fisher’s exact test. For samples that fit criteria for multiple grades, the highest grade was used. Inter-reader agreement was calculated as Cohen’s kappa (κ).

## Data Availability

RNA sequencing data have been deposited to dbGaP (Study Accession: phs002935.v1.p1, “Transcriptomic Profiling of Oropharyngeal Squamous Cell Carcinoma”). All other data are included in the article and/or Supplementary Materials.

## Supplementary Materials

The following supporting information can be downloaded at: https://www.mdpi.com/article/10.3390/cancers18183030/s1, **Figure S1**. HPV+ HNSCC TME signature enrichment scores across radiologic characteristics correlated with extranodal extension (ENE). Each boxplot represents the distribution of scores among patients positive for the indicated correlate by neuroradiologist review. Enrichment scores were calculated using single sample gene set enrichment analysis. The Mann–Whitney U test p values comparing ssGSEA scores for patients with and without each correlate are as follows: central hypodense area in node, *p* = 0.49, FDR = 0.68; indiscrete border, *p* = 0.52, FDR = 0.68; perinodal fat stranding, *p* = 0.12, FDR = 0.68; invasion into adjacent soft tissue, *p* = 0.98, FDR = 0.98; indiscrete borders of primary tumor, *p* = 0.53, FDR = 0.68; multiple involved lymph nodes, *p* = 0.62, FDR = 0.70; lobular contour, *p* = 0.39, FDR = 0.68; irregular nodal capsular enhancement, *p* = 0.32, FDR = 0.68; matted nodes, *p* = 0.39, FDR = 0.68.No feature was significantly associated with enrichment score (nominal *p* value > 0.05); **Table S1**: Association of report based iENE status with patient clinical characteristics; **Table S2**: Wheat2 signature genes; **Table S3**: Differentially expressed genes in radiology report-based iENE positive vs. radiology report-based iENE negative samples; **Table S4**: Differentially expressed genes between ML-ENE positive vs. ML-ENE negative samples; **Table S5**: Cox–model interaction analysis for iENE status and gene signature enrichment; **Table S6**: Extended data for RFS analysis.
