# Supplementary material for: Tumor Inflammation Confounds Radiographic Determination of Extranodal Extension in HPV-Associated Head and Neck Squamous Cell Carcinoma
Source: Cancers (Basel). 2026 Sep 18;18(18):3030. doi: 10.3390/cancers18183030 (PMC13605798; doi:10.3390/cancers18183030)
Supplement: Supplementary file 1 [file cancers-18-03030-s001.zip › Supplemental Figure S1.pdf]

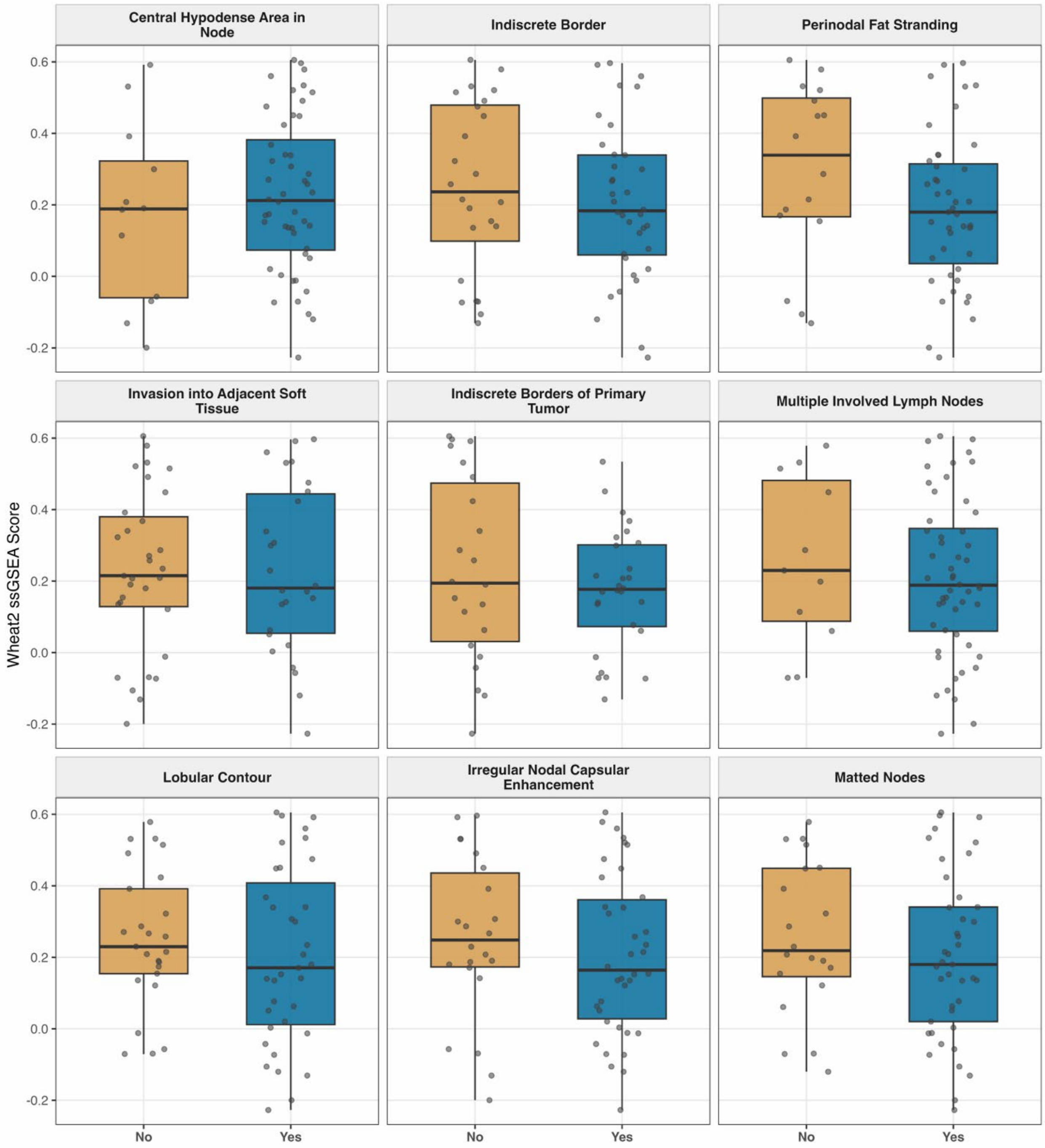

Figure S1. HPV+ HNSCC TME signature enrichment scores across radiologic characteristics correlated with extranodal extension (ENE). Each boxplot represents the distribution of scores among patients positive for the indicated correlate by neuroradiologist review. Enrichment scores were calculated using single sample gene set enrichment analysis. The Mann–Whitney U test p values comparing ssGSEA scores for patients with and without each correlate are as follows: central hypodense area in node, p

= 0.49, FDR = 0.68; indiscrete border, $p = 0.52$, FDR = 0.68; perinodal fat stranding, $p = 0.12$, FDR = 0.68; invasion into adjacent soft tissue, $p = 0.98$, FDR = 0.98; indiscrete borders of primary tumor, $p = 0.53$, FDR = 0.68; multiple involved lymph nodes, $p = 0.62$, FDR = 0.70; lobular contour, $p = 0.39$, FDR = 0.68; irregular nodal capsular enhancement, $p = 0.32$, FDR = 0.68; matted nodes, $p = 0.39$, FDR = 0.68.No feature was significantly associated with enrichment score (nominal p value > 0.05)
