# Supplementary material for: Tumor Inflammation Confounds Radiographic Determination of Extranodal Extension in HPV-Associated Head and Neck Squamous Cell Carcinoma
Source: Cancers (Basel). 2026 Sep 18;18(18):3030. doi: 10.3390/cancers18183030 (PMC13605798; doi:10.3390/cancers18183030)
Supplement: Supplementary file 1 [file cancers-18-03030-s001.zip › TableS1.pdf]

Table S1: Association of report based iENE status with patient clinical characteristics. *

iENE status	No	Yes	p-value
n	60	21	
Sex - n (% male)	56 (93.3)	20 (95.2)	1
Age in years - mean (SD)	56.84 (8.22)	54.72 (9.54)	0.33
<10 pack years smoking - n (%)	28 (45.1)	11 (52.4)	0.8
Primary site - n (%)			0.2
Tonsil	29 (48.3)	14 (66.7)	
Base of tongue	31 (51.7)	7 (33.3)	
AJCC 7 N-Stage - n (%)			0.086
N1	2 (3.3)	0 (0.0)	
N2	51 (85.0)	14 (66.7)	
N3	7 (11.7)	7 (33.3)	
AJCC7 T-STAGE – n (%)			0.091
T1	14 (23.3)	10 (47.6)	
T2	24 (40.0)	9 (42.9)	
T3	11 (18.3)	1 (4.8)	
T4	11 (18.3)	1 (4.8)	
Treatment type - n (%)			0.42
Chemoradiation	55 (91.7)	18 (85.7)	
Radiation alone	2 (3.3)	1 (4.8)	
Surgery + chemoradiation	3 (5.0)	1 (4.8)	
Unknown	0 (0.0)	1 (4.8)	
Neck dissection - n (%)			0.65
No neck dissection	40 (66.7)	14 (66.7)	
Primary treatment neck dissection	1 (1.7)	1 (4.8)	
Salvage neck dissection	16 (26.7)	6 (28.6)	
Salvage neck dissection w/ resection	3 (5.0)	0 (0.0)	

**T-test was used to determine significance for age, and fishers exact test was used for all other variables. No samples in this archival cohort had pENE data available.*
